# The effect of antimicrobial photodynamic therapy on periodontal disease and glycemic control in patients with type 2 diabetes mellitus

**DOI:** 10.1007/s00784-023-05239-0

**Published:** 2023-09-06

**Authors:** Sara Brinar, Aleš Skvarča, Boris Gašpirc, Rok Schara

**Affiliations:** 1 Department of Oral Medicine and Periodontology, University Medical Center Ljubljana, Ljubljana, Slovenia; 2 Community Health Centre Murska Sobota, Murska Sobota, Slovenia; 3 Department of Endocrinology, Diabetes and Metabolic Diseases, University Medical Centre Ljubljana, Ljubljana, Slovenia; 4 Department of Oral Medicine and Periodontology, Faculty of Medicine, University of Ljubljana, Ljubljana, Slovenia

**Keywords:** Type 2 diabetes mellitus, Periodontal disease, Antimicrobial photodynamic therapy, Indocyanine green

## Abstract

**Objectives:**

This study is aimed at determining the effect of concomitant antimicrobial photodynamic therapy (aPTD) on periodontal disease and glycaemic control in patients with type 2 diabetes mellitus (T2DM).

**Materials and methods:**

Twenty-four patients with T2DM were enrolled in the study. Periodontal clinical parameters were assessed by measuring probing pocket depth (PPD), clinical attachment loss (CAL), gingival recession (GR), full-mouth bleeding score (FMBS), full-mouth plaque score (FMPS), and full-mouth sulcus bleeding score (FMSBS). Glycated haemoglobin A1c (HbA1c) was measured. To determine the presence of the following periodontal pathogenic bacteria, *Aggregatibacter actinomycetemcomitans*, *Prevotella intermedia*, *Porphyromonas gingivalis*, *Tannerella forsythia*, and *Treponema denticola*, subgingival plaque samples were taken from two periodontal pockets per jaw with the greatest PPD using paper tips. Patients were randomly divided into the test and control group. In the test group, full-mouth disinfection was performed in combination with aPTD. In the control group, only full-mouth disinfection was performed.

**Results:**

The results showed an improvement in periodontal clinical parameters in both groups. The difference between the groups in favour of the test group was statistically significant for BOP. The HbA1c level decreased in both groups. The difference was not statistically significant. The results of the microbiological analysis suggest that the presence of periodontal pathogenic bacteria is lower with additional antimicrobial photodynamic therapy with statistically significant difference for *T. forsythia*.

**Conclusions:**

Additional aPDT causes a significant reduction in BoP in the proportion of positive sites for periodontal pathogens.

**Trial registration:**

ClinicalTrials.gov ID: NCT05816941.

**Clinical relevance:**

aPTD is a noninvasive adjunctive therapy that can positively influence the periodontal treatment outcome.

## Introduction

Diabetes is a chronic metabolic disease characterised by elevated blood glucose levels, leading to severe damage to the heart, blood vessels, eyes, kidneys, and nerves [1]. About 90% of patients have diabetes mellitus type 2. Most patients are overweight and have an increased body fat percentage, especially in the abdominal region. The World Health Organisation (WHO) estimated the number of diabetics in 1985 at 30 million people worldwide, and this number rose to 217 million in 2005. By 2030, WHO predicts that this number will rise to at least 366 million [2].

Periodontal disease is characterised by chronic inflammation of the periodontal tissues caused by the accumulation of dental biofilm. After plaque removal, gingivitis is a reversible condition. However, long-term biofilm accumulation leads to irreversible loss of periodontal tissue (connective tissue, periodontal ligament, and alveolar bone) [3, 4]. Because of its chronic inflammatory nature and multifactorial complexity, periodontal disease has also been associated to several systemic diseases and conditions such as cardiovascular diseases, diabetes mellitus, and obesity [5, 6].

Numerous studies confirm that diabetes mellitus increases the risk of gingivitis, periodontitis, and implant complications [7–9]. However, periodontal disease also impairs glycaemic control in people with diabetes mellitus via inflammatory mediators [2, 10, 11]. Periodontal disease represents the sixth complication of diabetes mellitus [1]. Epidemiological studies have shown that the risk of alveolar bone loss and loss of attachment level is three times higher in people with diabetes and poor metabolic control [12, 13]. Nevertheless, there is no increased risk of periodontal disease in people with good metabolic control [14].

Systemic diseases can increase the risk of periodontitis or affects its progression by increasing the inflammatory burden and also by causing alterations to the local microbiome, which can become more pathogenic [15, 16]. There is still a shortage of human research data about significant differences in the microbiological composition of the biofilm in people with and without diabetes [17], suggesting that an altered immune response plays an important role in the development of periodontal disease. Diabetes impairs neutrophil granulocyte adherence, chemotaxis, and phagocytosis, which increase periodontal tissue destruction [2, 18, 19]. It also causes an exaggerated and prolonged inflammatory response, impaired wound healing, and more severe diabetes-associated periodontal disease [20].

The success of periodontal treatment is based on the reduction of periodontal pathogenic bacteria in the dental biofilm and other ecological niches in the oral cavity [21]. Mechanical debridement, scaling, and root planing (SRP) is the treatment of choice for most periodontal infections [22]. Removal of subgingival biofilm results in a microbiological shift that improves the clinical parameters of periodontitis [23]. Standard periodontal treatment is based on mechanical quadrant debridement, scaling, and root planing and is usually completed after 4 to 6 weeks. Quirynen et al. presented a full-mouth disinfection protocol in which non-surgical periodontal treatment is completed in one session or two sessions within 24 h [24]. The aim is to minimise the risk of recolonization of previously treated pockets with pathogens from untreated sites or other oral niches, such as the tongue or tonsils [25].

With mechanical debridement, it is not always possible to completely eliminate the bacteria that cause periodontal infection, which is why residual pockets that bleed upon probing may still be present. Repeated scaling and root planning can lead to tooth structure loss [26], gingival recession [27], and tooth hypersensitivity [28]. Therefore, antimicrobial photodynamic therapy (aPDT) can be an efficient adjunct to standard periodontal treatment [29]. aPDT is based on the principle that light of an appropriate wavelength can stimulate the photosensitizer to produce singlet oxygen and other highly reactive free radicals, which are highly toxic to bacteria and their products [30]. A number of photosensitizers, including methylene blue and toluidine blue, have been investigated with lasers of different wavelengths for periodontal therapy [31]. Indocyanine green (ICG) is one of the photosensitizers commonly used in soft tissue surgery, liver function tests, ophthalmology, and oncology. Recently, it has gained special attention in dentistry [32]. Its absorption peak is near the emission maximum of the available dental diode laser (at 800 nm) [33]. The activation of ICG and the resulting release of free radicals can also occur in the absence of oxygen [34]. This can be of great benefit in the anaerobic conditions found in deep periodontal pockets [35]. Boehm and Ciancio found rapid and significant uptake of ICG by periodontal pathogens, which resulted in significant reduction of *A. actinomycetemcomitans* and *P. gingivalis* after activation with an 810-nm diode laser [36]. The study by Monzavi et al. showed that ICG with an 810-nm diode laser in combination with SRP leads to a complete resolution of inflammation and a significant reduction in periodontal pockets [30].

## Materials and methods

This study was a randomised single-blinded controlled clinical trial. The study was performed in accordance with the ethical standards as laid down in the 1964 Declaration of Helsinki. The protocol was approved by the National Medical Ethics Committee of the Republic of Slovenia (No: 0120–539/2015–2). A sample size of 12 patients per group was set to exceed a power of 80% (10 patients per group) to detect the difference of 1 mm in clinical attachment loss (CAL) and 1% glycated haemoglobin (HbA1c) between the groups with predicted values of CAL 3.5 mm and a variability of 1.3 mm and the groups with predicted values of HbA1c 8.0% and a variability of 1.5%.

The subjects with type 2 diabetes mellitus were referred from the Department of Endocrinology, Diabetes and Metabolic Diseases of the University Medical Centre Ljubljana, to the Department of Dentistry and Periodontology of the University Medical Centre Ljubljana. Detailed dental and medical records were obtained. The inclusion criteria were as follows: age between 40 and 75 years, diabetes mellitus type 2 with an HbA1c value > 7.0%, at least ten teeth in the maxilla and mandible, and at least four teeth with a probing pocket depth ≥ 5 mm and bleeding on probing. The exclusion criteria were as follows: antibiotic treatment in the last 4 months, periodontal treatment in the last 6 months, and any change in antihyperglycaemic treatment 3 months prior to participation. Pregnant and lactating women, smokers, and former smokers who had stopped smoking less than 5 years before participation were also excluded. Subjects who met the inclusion criteria were invited to participate in the study. Each subject was informed in detail about the benefits and risks of the treatment and signed the informed consent form before participation.

### Clinical parameters, microbiological assessment, and glycaemic monitoring

At baseline and 90 days after treatment a full periodontal examination was conducted, and the following periodontal clinical parameters were assessed:

- Probing pocket depth (PPD) was measured from the gingival margin to the base of the clinical pocket using manual probe at six sites around each tooth.
- Clinical attachment level (CAL) was calculated as a distance in millimetres from a cemento-enamel junction (CEJ) or the border of cervical a restoration to the bottom of the probable pocket at six sites per tooth. Two measurements were used to calculate the CAL: the PPD and the distance from the gingival margin to the CEJ.
- Gingival recession (GR) was measured from the CEJ to the gingival margin using manual probe at six sites around each tooth.
- Full-mouth bleeding score (FMBS) was measured at six sites around each tooth with the PPD measurements based on the presence or absence of bleeding up to 30 s after probing. The percentage of bleeding sites was calculated per subject per visit.
- Full-mouth plaque score (FMPS) was calculated as the percentage of tooth surfaces with the presence of plaque at six sites per tooth. Plaque was detected by the use of a periodontal probe.
- Full-mouth sulcus bleeding score (FMSBS) was calculated as the percentage of tooth surfaces with the presence of sulcular (marginal) bleeding per tooth. Sulcus bleeding was detected after gentle passing of tip of the periodontal probe along gingival margin at six sites per tooth.

All measurements were performed by a single experienced clinician (SB) without information about allocated treatment (blind), and the intra-examiner calibration value was 0.89. All clinical measurements were performed with a manual probe (Williams SE, PCP10, Hu-Friedy).

Pooled subgingival plaque samples were taken from two deepest periodontal pockets in each jaw at baseline and 90 days after treatment. Plaque samples were collected with sterile paper tips after supragingival soft and hard debris had been removed according to the manufacturer’s instructions. Paper tip was inserted to the base of the periodontal pocket for 30 s and put into Eppendorf tube with 1.5 ml of reduced transport medium. All samples were put into transporting box filled with ice and transported to the Microbiology Laboratory (Institute of Microbiology, Faculty of Medicine, University of Ljubljana, Slovenia) for analysis performed within 4 h after sampling. A commercially available micro-Ident test (Hain Lifescience, Nehren, Germany) was used for this purpose. The presence of five periodontal pathogens, *Aggregatibacter actinomycetemcomitans* (AA), *Porphyromonas gingivalis* (PG), *Prevotella intermedia* (PI), *Tannerella forsythia* (TF), and *Treponema denticola* (TD), was determined by polymerase chain reaction (PCR) followed by hybridization against species-specific DNA probes. According to the manufacturer, the cut-off of the test is set at 10^3^ to 10^4^ genome equivalents [37].

For HbA1c analysis, blood samples were collected for each subject at baseline and 90 days after treatment. The analysis was performed at the Department of Endocrinology, Diabetes and Metabolic Diseases, University Medical Centre Ljubljana.

### Treatment protocol

After full periodontal examination, all patients received nonsurgical periodontal therapy. All patients received motivation and instruction in proper oral hygiene followed by a full-mouth disinfection based on original approach described by Quirynen et al. in 1995 [24]. Supra- and subgingival hard and soft deposits were removed using ultrasonic instrument (PiezoLED ultrasonic scaler with Piezo Scaler tip 203, KaVo dental, Germany), after ultrasonic instrumentation scaling and root planing of sites with PD ≥ 5 mm were performed under local anaesthesia (Ultracaine D-S, Sanofi, France) using Gracey curettes (Hu-Friedy, USA) in all 4 quadrants (up to one hour per quadrant). After removal of hard and soft deposits, pocket irrigation with sterile 0.2% chlorhexidine digluconate (chlorhexidine digluconate, Pharmacy of University medical centre Ljubljana, Slovenia), three times in 10 min and brushing of the dorsum of the tongue for 60 s with chlorhexidine-digluconate 0.2% gel (Curasept ADS 720, Curasept, Italy) was performed. Patients were instructed to rinse mouth with sterile 0.2% chlorhexidine digluconate (chlorhexidine digluconate, Pharmacy of University medical centre Ljubljana, Slovenia) twice for 1 min (gargling during the last 10 s to reach the tonsils).

Afterwards, the patients were randomly divided into test and control groups by computer randomisation list (excel, Microsoft, USA). The patients in the test group received aPDT as adjuvant treatment in pockets with PPD ≥ 5 mm. For this purpose, a diode laser (Fotona XD -2, Fotona,, Slovenia) with a wavelength of 810 nm, a power of 250 mW, and the photosensitizing agent indocyanine green (Cardiogreen, Sigma-Aldrich, USA) at a concentration of 1 mg/ml was used. First, the area to be irradiated was isolated, and the photosensitizing agent was applied to the periodontal pocket. After 60 s, the supragingival excess of the photosensitizing agent was removed by gentle rinsing with a saline solution. Ten-second irradiation of each site followed.

All patients were instructed about postsurgical care, pain management if needed, and mouth rinsing with chlorhexidine 0.12% (Curasept ADS 212, Curasept, Italy) mouth wash twice daily for 1 min for 14 days after treatment.

Ninety days after the treatment, full periodontal examination, microbiological sampling, and blood sampling for HbA1c analysis were performed, and the data was analysed.

### Statistical analysis

The statistical analysis was carried out with the programme Statistica 10, StatSoft Inc. The non-parametric repeated measures test ANOVA and the Newman-Keuls test were applied to compare the means of periodontal and microbiological parameters and glycaemic control. Statistical significance was defined at *p* < 0.05.

## Results

Twenty-four patients participated in this three-month study. The healing process was smooth, and none of the participants reported any adverse effects such as discolouration of the teeth, gums, or mucous membranes.

**Fig. 1:**
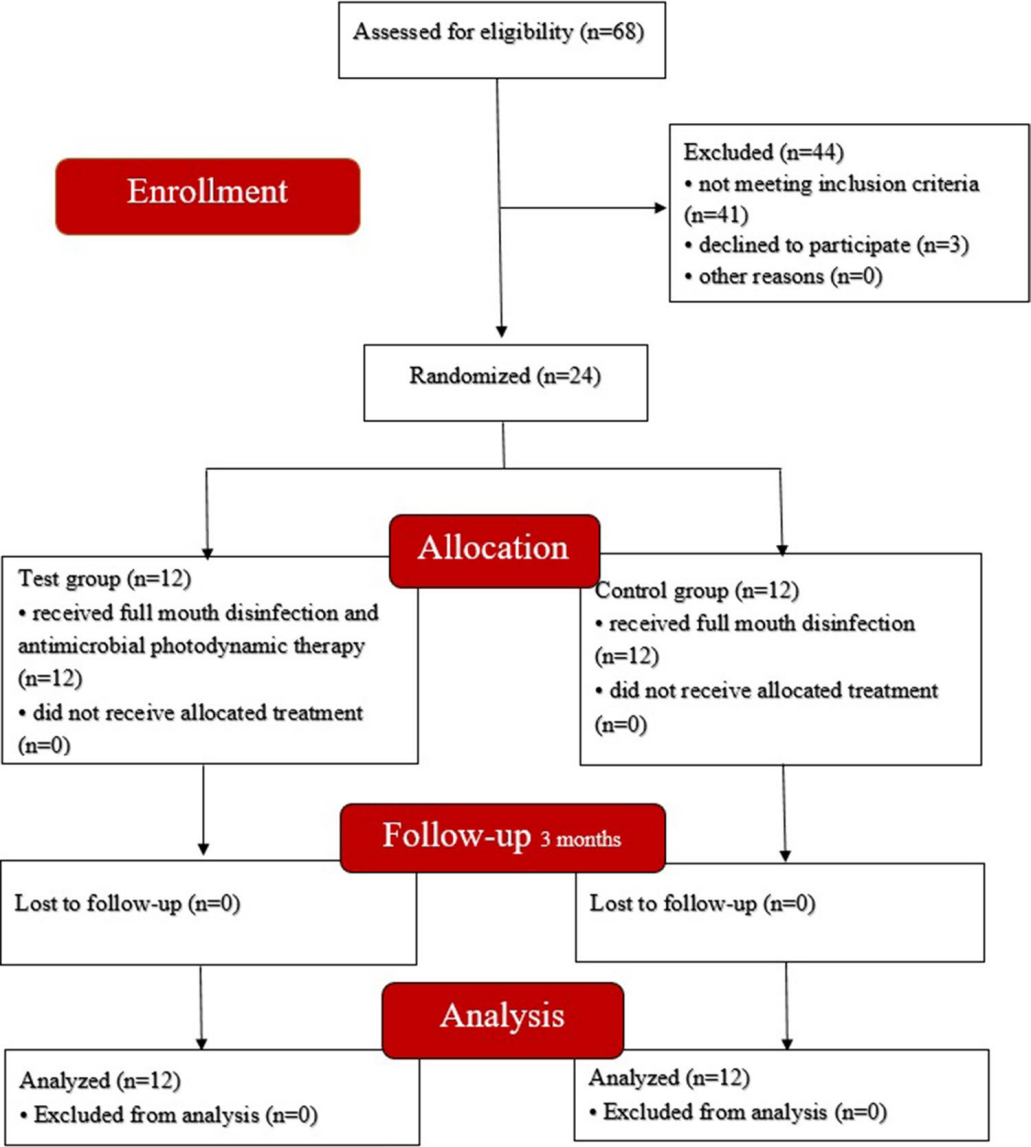
Consort flow diagram

Figure 1 shows the flow chart of the study. Twenty-four patients were randomised into the test and control groups. The demographic characteristics are shown in Table 1. Analysis showed that demographic characteristics, periodontal indices, and initial clinical and glycaemic parameters were homogeneous between the two groups, with no statistically significant differences (*p* > 0.05).

**Table 1:** Demographic characteristics of the test and control groups (*T*, test group; *C*, control group; *p*, statistical significance; *HbA1c*, glycated haemoglobin; *NS*, not significant)

	T	C	*p*
Number (*n*)	12	12	NS
Gender (m/f)	7/5	6/6	NS
Average age (range) (years)	63 (47–73)	67.5 (56–75)	NS
HbA1c (%)	7.9 ± 0.3	8.2 ± 0.3	NS
Duration of DM (years)	12.8 ± 6.0	18.7 ± 7.5	NS

### Periodontal clinical parameters

At baseline, there were no statistically significant differences between the two groups in the clinical periodontal parameters (PPD-, CAL, GR, FMBS, FMPS, and FMSBS). Both groups showed an improvement in periodontal clinical parameters, as shown in Table 2. In addition, there was a clinical attachment gain in both groups (0.5 ± 0.9 mm in the test group and 0.4 ± 0.9 mm in the control group, not shown), but the difference between the groups was not statistically significant (*p* > 0.05).

**Table 2:** The periodontal clinical parameters in the test (T) and control (C) groups at baseline (T0) and after three months (T3)

		T		C
	T0	T3	T0	T3
PPD (mm)	3.3 ± 0.2	*2.6 ± 0.2	3.1 ± 0.2	*2.7 ± 0.1
CAL (mm)	3.7 ± 0.02	*3.2 ± 0.2	3.6 ± 0.2	†3.2 ± 0.2
GR (mm)	0.5 ± 0.03	†0.43 ± 0.03	0.46 ± 0.02	0.47 ± 0.03
FMBS (%)	21.8 ± 4.2	†7.1 ± 3.1	26.6 ± 4.2	†‡19.1 ± 3.1
FMSBS (%)	31.1 ± 8.5	†11.2 ± 5.2	29.6 ± 8.5	†15.9 ± 5.2
FMPS (%)	48.4 ± 7.3	†34 ± 7.4	47.7 ± 7.3	†34.7 ± 7.4

*Statistically significant (*p* < 0.001) intragroup difference, †statistically significant (*p* < 0.05) intragroup difference, ‡statistically significant (*p* < 0.05) intergroup difference

The comparison between the groups showed a statistically significant difference at FMBS (*p* < 0.05). However, for PPD, CAL, GR, FMSBS, and FMPS, the difference was not statistically significant (*p* > 0.05). Table 3 shows frequency distribution of CAL gains and residual PPD in both groups.

**Table 3:** Frequency distribution of CAL gains and residual PPD in treatment and control groups after 3 months

	T				C			
	CAL gain		Residual PPD		CAL gain		Residual PPD	
	*n*	%	*n*	%	*n*	%	*n*	%
0–1 mm	517	72.9	50	3.3	363	66.6	27	1.9
2–3 mm	176	24.8	1309	85.9	161	29.5	1142	82.4
4 mm	13	1.8	80	5.2	16	2.9	128	9.2
5 mm	1	0.1	49	3.2	5	0.9	65	4.7
≥ 6 mm	2	0.2	36	2.4			24	1.7

### Microbiological analysis

At baseline, the analysis showed no statistically significant differences between the groups (*p* > 0.05). In the test group, the proportion of positive sites for *P. gingivalis*, *P. intermedia*, *T. denticola*, and *T. forsythia* decreased significantly (*p* < 0.05). The proportion of *A. actinomycetemcomitans* was 0% at baseline and after 180 days. The proportion of positive sites for *A. actinomycetemcomitans*, *P. gingivalis*, *T. denticola*, and *T. forsythia* decreased in the control group. However, the difference was not statistically significant (*p* > 0.05). The proportion of positive sites for *P. intermedia* did not change.

The comparison between the groups after three months showed a statistically significant difference in the proportion of positive sites for *T. forsythia* in favour of the test group (*p* < 0.05).

**Fig. 2:**
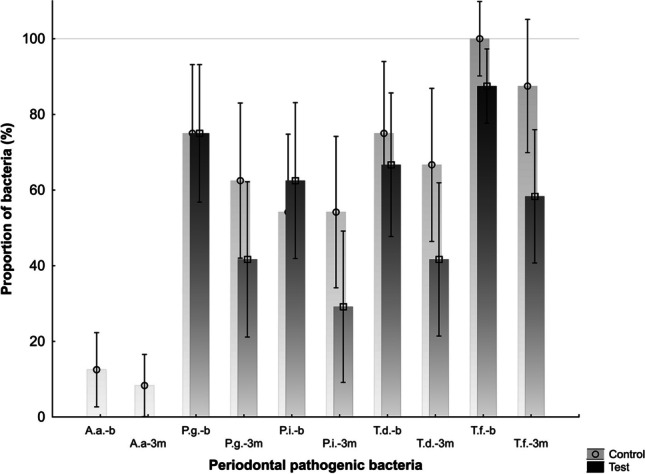
The proportion of periodontal pathogenic bacteria in the test (T) and the control (C) groups on the initial exam and after 3 months (A.a., *Aggregatibacter actinomycetemcomitans*; P.g., *Porphyromonas gingivalis*; P.i., *Prevotella intermedia*; T.d., *Treponema denticola*; T.f., *Tannerella forsythia*; PP, baseline; K, 3 months; ZDR, test group; K, control group)

Table 4 shows the proportions of positive sites for periodontal pathogens. Figure 2 shows the comparison of the proportion of positive sites for the test and control groups at baseline and after 3 months.

**Table 4:** The proportion of periodontal pathogenic bacteria in the test (T) and control (C) groups at baseline (T0) and after 3 months (T3) (*A.a.*, *Aggregatibacter actinomycetemcomitans*; *P.g.*, *Porphyromonas gingivalis*; *P.i.*, *Prevotella intermedia*; *T.d.*, *Treponema denticola*; *T.f.*, *Tannerella forsythia*)

Group	T		C	
Pathogen	T0	T3	T0	T3
*A.a*	0	0	12.5 ± 4.8	8.3 ± 4
*P.g*	75 ± 9	*41.7 ± 10.2	75 ± 9	62.5 ± 10.2
*P.i*	62.5 ± 10.2	*29.2 ± 10	54.2 ± 10.2	54.2 ± 10
*T.d*	66.7 ± 9	*41.7 ± 10	75 ± 9.4	66.7 ± 10
*T.f*	87.5 ± 4.9	+ *58.3 ± 8.7	100 ± 4.9	87.5 ± 8.7

*Statistically significant (*p* < 0.05) intergroup difference; + statistically significant (*p* < 0.05) intergroup difference after 3 months

### Glycaemic control

**Fig. 3:**
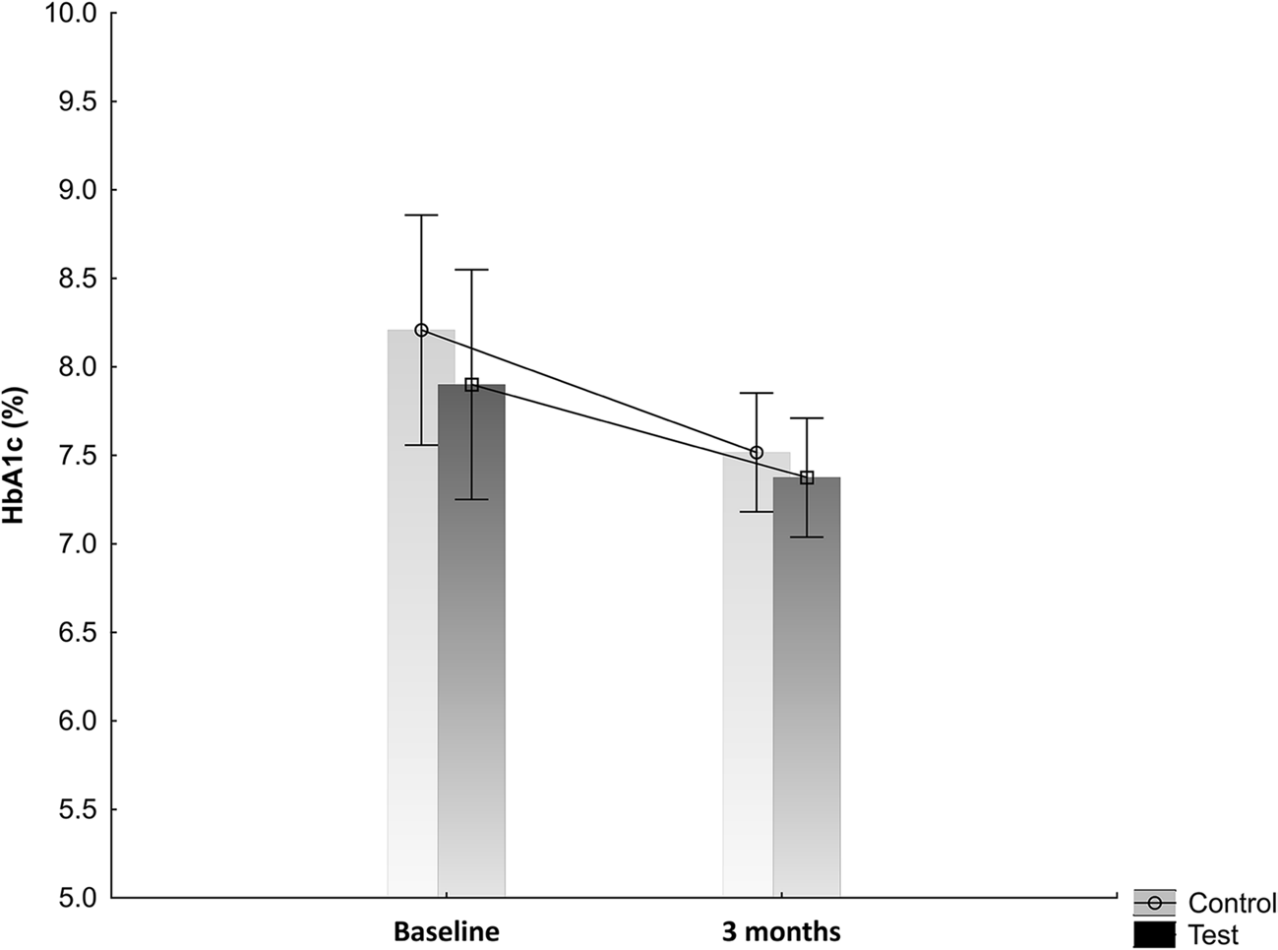
The level of HbA1c in the test and the control groups at baseline and after 3 months

At baseline, there was no significant difference between the groups’ levels of glycated haemoglobin (HbA1c). The HbA1c level decreased from 7.9 ± 0.3% to 7.4 ± 0.2% in the test group and from 8.2 ± 0.3% to 7.5 ± 0.2% in the control group. However, the comparison between the groups showed no statistically significant difference after 3 months. Figure 3 shows the HbA1c value in the test and control groups at baseline and after 3 months.

## Discussion

Recently, numerous studies have investigated the effect of adjunctive antimicrobial photodynamic therapy in the treatment of periodontal disease. Some studies have shown additional benefits after combined treatment with non-surgical periodontal therapy and aPDT [38–40], while others have shown no benefit of combined treatment [41–43]. Controversy in these results could be due to a wide variety between protocols, differences between groups and severity of periodontitis, and different study designs [44]. Methylene blue and toluidine blue are most commonly used as photosensitizers, combined with diode lasers with a wavelength of 635 to 675 nm [38, 39, 41, 42]. Only a few clinical studies investigated the effect of the photosensitizer indocyanine green in combination with a diode laser with a wavelength of 810 nm for the treatment of periodontal disease [30, 35, 45]. Most studies investigated the effect of combined non-surgical periodontal therapy and aPDT in healthy individuals, but only a few in patients with T2DM [44, 46–48]. To our knowledge, this is the first clinical study to investigate the effect of concomitant aPDT with the use of indocyanine green as a photosensitizer and a diode laser with a wavelength of 810 nm on periodontal treatment in patients with T2DM.

Our study showed an improvement in periodontal clinical parameters in both groups. In addition, both groups showed a similar reduction in probing pocket depth (PPD) and a similar increase in clinical attachment level. However, when comparing the groups, the difference was not statistically significant. This result was comparable to two other studies [44, 47] that investigated the effect of adjunctive aPDT in patients with T2DM and used methylene blue as a photosensitizer and a diode laser with wavelengths of 660 nm and 670 nm. The results of both studies showed a statistically significant reduction in PPD, CAL, and a reduction in bleeding on probing (BoP). However, the comparison between the groups did not show statistically significant differences. Barbosa et al. in their 2018 study showed a statistically significant improvement in periodontal clinical parameters (PPD, CAL, and BOP) after 30 days [46]. The level of PPD and CAL remained stable in both groups after 3 and after 6 months; however, the level of BOP remained stable only in the test group with combined non-surgical periodontal treatment and aPDT. In the control group, the baseline level of BOP and BOP was statistically insignificant after 3 and after 6 months. The comparison between the groups showed no statistically significant differences. In patients with poorly controlled diabetes, it is important to emphasise that an impaired immune system, vascular complications, and impaired wound healing alter the response to conventional periodontal treatment and also to the use of aPDT [44, 46]. The limitation in comparing the above studies is using different photosensitizers and diode lasers with different wavelengths.

However, a few studies showed a statistically significant reduction in BOP [43, 49], which is consistent with our results. In 2016, Monzavi et al. also showed a statistically significant reduction in BOP [30]. They used indocyanine green as a photosensitizer and a diode laser with a wavelength of 810 nm. They discovered a statistically significant PPD, BOP, and sulcular bleeding index (SBI) reduction in the test group combining non-surgical periodontal therapy and aPDT. Comparison between groups showed no statistically significant differences at CAL. The limitation compared to this study is that the patients do not have T2DM.

Numerous studies have confirmed that diabetes mellitus increases the risk of periodontal disease and that, on the other hand, periodontal disease impairs glycaemic control and increases the risk of complications of diabetes [2]. Inflamed periodontal tissues serve as a source of the following inflammatory mediators: TNF-α, IL-6, and IL-1, which affect glucose and lipid metabolism and act as insulin antagonists [2, 50]. Chronic gramme-negative infection of periodontal origin can cause insulin resistance and contributes to hyperglycaemia and complications of diabetes [50]. Many researchers have attempted to determine the effects of periodontal treatment on glycaemic control, and some showed positive effects [51–53], while others did not [54–56].

In our study, both groups showed a reduction in HbA1c, but the difference between the groups was not statistically significant.

Furthermore, few studies investigated the effect of concomitant aPDT on glycaemic control. The 2016 “split-mouth” study by Castro dos Santos et al. showed no statistically significant effect of aPDT on glycaemic control [44]. In 2018, Barbosa et al. compared SRP and SRP + aPDT; however, there was no statistically significant difference in glycaemic control between the groups [39]. In 2009, Al-Zahrani et al. compared three treatment modalities: SRP, SRP + aPDT, and SRP + doxycycline. All three groups showed a reduction in HbA1c, but the difference was statistically significant only for the SRP + doxycycline group [47]. This reduction in HbA1c can be explained by the mechanism of action of the antibiotic, which acts on the organism by inhibiting matrix metalloproteinase synthesis and inhibiting non-enzymatic glycation, which can also affect haemoglobin glycation [57]. Our research findings are consistent with the studies mentioned above, which also failed to demonstrate any benefits of complementary aPDT for glycaemic control.

To the best of our knowledge, our study is the first to evaluate microbiological parameters when investigating the effect of supplemental aPDT in patients with T2DM. The results showed a statistically significant reduction in the proportion of positive sites for *P. gingivalis*, *P. intermedia*, *T. denticola*, and *T. forsythia* (*p* < 0.05) in the test group from baseline to 3 months after treatment. The proportion of positive sites for *A. actinomycetemcomitans* was 0% at baseline and after 3 months. In the control group, the reduction in the proportion of positive sites for *A. actinomycetemcomitans*, *P. gingivalis*, *T. denticola*, and *T. forsythia* was not statistically significant (*p* > 0.05). The protocol of complete mouth disinfection in the control group did not affect the proportion of positive sites for *P. intermedia*.

The low prevalence of *A. actinomycetemcomitans* in the test and control groups can be explained by the fact that *A. actinomycetemcomitans* is more common in younger patients than in older periodontitis patients [58]. The mean age of our patients was 63 years in the test group and 67.5 years in the control group.

The comparison between the groups showed a statistically significant reduction in the proportion of positive sites for *T. forsythia* in favour of the test group (*p* < 0.05). A significant reduction in the proportion of positive sites for *T. forsythia* could be the cause of a significant reduction in FMBS in the test group. In addition, *T. forsythia* was detected more frequently and in higher numbers in the active periodontal lesions [59, 60]. The association between reduced BoP and reduced proportion of *T. forsythia* was found after SRP [60, 61].

The results of clinical studies on the effect of adjunctive aPDT on periodontal pathogenic bacteria are contradictory. In 2015, Moreira et al. compared SRP and SRP + aPDT and showed a statistically significant reduction of four periodontal pathogens (*P. gingivalis*, *T. forsythia*, *T. denticola*, *A actinomycetemcomitans*) in the test group after 3 months [62]. In 2012, Theodoro et al. achieved similar results and significantly reduced five periodontal pathogens (*A. actinomycetemcomitans*, *P. gingivalis*, *T. forsythia*, *T. denticola*, and *P. intermedia*) in the group with additional aPDT after 6 months [63]. On the other hand, Polansky et al. in 2009 found no advantages of adjunctive aPDT compared to non-surgical periodontal treatment alone and showed no statistically significant differences in microbiological parameters [42].

As mentioned above, few clinical studies investigated the effect of additional aPDT using ICG as a photosensitizer. In an in vitro study, Boehm and Ciancio found rapid and significant uptake of ICG into periodontal pathogens and activation with an 810-nm diode laser resulted in significant destruction of *A. actinomycetemcomitans* and *P. gingivalis* [36]. In their clinical trial of adjunctive aPDT using ICG as a photosensitizer, Srikanth et al. showed a more significant reduction in periodontal pathogens than SRP [45]. The results of our study show a more significant reduction in the proportion of positive sites for periodontal pathogens after adjunctive use of aPDT. The study indicates that further investigation is needed with a larger number of participants.

It is worth noting that it is difficult to compare our study with the studies mentioned above because diode lasers with different wavelengths, different photosensitizers, different participant groups, and different treatment protocols were used. Most studies used methylene blue or toluidine blue as photosensitizers, and only a few used ICG. Its absorption peak is near the emission maximum of the available dental diode laser (at 800 nm) [33], while the absorption peak of methylene blue and toluidine blue is between 635 and 670 nm [64]. The effect of ICG is mainly photothermal (80% photothermal, 20% photochemical) [64]. The photothermal and photochemical effects of ICG make this photosensitizer significant for eradicating pathogens in deep periodontal pockets, furcations, or invaginations. Therefore, ICG with an 810-nm diode laser can be considered a promising candidate for adjunctive periodontal treatment [30].

## Conclusion

This study is aimed at determining the influence of concomitant aPDT using ICG as a photosensitizer and a diode laser with a wavelength of 810 nm on periodontal disease, glycemic control, and microbiological parameters in patients with T2DM. We discovered a statistically significant reduction in FMBS in favour of the test group; however, the difference in other periodontal parameters (PPD, CAL, GR, FMSBS, and FMPS) was not statistically significant. Both groups showed a reduction in HbA1c, but the difference between the groups was not statistically significant. In the short term, complementary aPDT does not contribute to better glycaemic control. The results of our study suggest that the effect of supplementary aPDT on reducing the proportion of positive sites for periodontal pathogens is more significant than the protocol of complete oral disinfection alone.

We can conclude that the additional aPDT does not contribute to better glycaemic control. However, it causes a more significant reduction in FMBS and in the proportion of positive sites for periodontal pathogens. A long-term study with a larger number of participants would be needed to confirm our results.

## Data Availability

All Research data will be available or shared on individual request. Example: conducting Meta-Analysis.
